# The Association between Within-Visit Blood Pressure Variability and Carotid Artery Atherosclerosis in General Population

**DOI:** 10.1371/journal.pone.0097760

**Published:** 2014-05-16

**Authors:** Yan Li, Jing Liu, Wei Wang, Dong Zhao

**Affiliations:** Department of Epidemiology, Beijing An Zhen Hospital, Capital Medical University, Beijing Institute of Heart, Lung and Blood Vessel Diseases, Beijing, China; University of Perugia, Italy

## Abstract

**Objectives:**

To determine whether within-visit blood pressure (BP) variability based on three measurements over minutes is associated with increased carotid intima-media thickness (IMT) and plaque in a general population.

**Methods:**

A cross-sectional survey was performed in 2007, and a total of 1222 Beijing community residents aged 50–79 years belonging to part of the Chinese Multi-Provincial Cohort Study (CMCS) were recruited in this study. BP was measured three times at 5-minute intervals during a single visit, and the maximum absolute difference (MAD) between any two readings of three measurements was used to indicate within-visit BP variability. Carotid IMT and plaque scanned by B-mode ultrasound were identified as the surrogate end points in the intermediate stage of atherosclerosis.

**Results:**

After adjustment for established cardiovascular risk factors, the odds ratio (OR) (95% confidence interval (CI)) for increased carotid IMT and internal carotid plaque associated with the highest within-visit diastolic BP (DBP) variability (MAD > mean + standard deviation (SD)) compared with participants in the lowest within-visit DBP variability (MAD ≤ mean −SD) was 4.92 (1.48–16.42) and 6.07 (1.31–28.10), respectively, in the normotensives (*P* = 0.01; *P* = 0.02). The OR (95% CI) for internal carotid plaque associated with the highest within-visit systolic BP (SBP) variability (MAD >mean +SD) compared with participants in the lowest within-visit SBP variability (MAD ≤ mean −SD) was 3.54 (1.26–10.00) in the hypertensives on antihypertensive therapy (*P* = 0.02).

**Conclusions:**

Within-visit DBP variability was associated with increased carotid IMT and internal carotid plaque in the normotensive population, and within-visit SBP variability was associated with internal carotid plaque in hypertensive patients undergoing antihypertensive therapy.

## Introduction

High blood pressure (BP) is a major predictor of stroke and other vascular events, accounting for approximately 54% of stroke and 47% of ischemic heart disease worldwide [1]. The conventional notion is generally accepted that the average value of BP based on multiple measurements taken during one or multiple visits is the most important determinant of blood-pressure-related risk for vascular events [2], [3], [4]. However, because BP is an inherently variable physiologic feature, the traditional perception of hypertension has been challenged by new findings from several recent studies demonstrating that visit-to-visit BP variability might be an important risk factor for stroke and all-cause mortality [5], [6], [7]. In these studies, within-visit BP variability was described as either a weak or insignificant predictor of vascular events [5], [6]. In contrast to visit-to-visit BP variability, within-visit BP variability obtained during a single visit as an indicator reflecting a transient fluctuation of BP over a very short period has been applied more to evaluate the variation in emotional and sympathetic activity, with previous research showing a link between a highly active sympathetic nervous system and increased cardiovascular risk [8], [9]. Although a possible potential mechanism had been reported whereby increased variability in BP might increase intra-vascular pressure or shear force, subsequently causing endothelial function damage and promoting the development of atherosclerosis [10], [11], it has remained uncertain whether within-visit BP variability plays a role in the process of atherosclerosis.

Carotid artery atherosclerotic lesions scanned by B-mode ultrasound are commonly used as a surrogate end point of vascular outcomes in epidemiological studies aimed at assessing preclinical atherosclerosis, and increased carotid IMT and plaque may be attributable to either functional or structural changes in the vessel wall, or both. To our knowledge, there have been few studies of the relationship between within-visit BP variability and carotid IMT and plaque. Moreover, the available data showed an inconsistency of relevance of within-visit BP variability to cardiovascular events and mortality [5], [12]. We designed this study to determine whether within-visit BP variability derived from three measurements in the sitting position over minutes during a single visit is associated with carotid IMT and plaque. The aim is to elucidate the effect of transient BP variability at several-minute intervals, in the hope of promoting the application of within-visit BP variability as a convenient measure for the assessment of preclinical atherosclerosis. Consequently, this technique may have clinical implications for the early prevention of atherosclerotic cardiovascular diseases.

## Methods

### Study population

The Chinese Multi-Provincial Cohort Study (CMCS) was conducted in 11 major provinces in China, including Beijing, from 1992 to date. The details of CMCS have been reported in previous studies [13]. Our study enrolled Beijing community residents selected from part of the CMCS cohort. A cross-sectional survey of cardiovascular risk factors and carotid ultrasound examination was performed by trained investigators and sonographers in 2007 from September to November. We invited 1982 residents of the Peking University community from the CMCS for this examination, and a total of 1255 subjects responded. Of them, 33 subjects were excluded for a previously diagnosed history of cardiovascular disease; the remaining 1222 participants aged 50–79 years were finally confined in this study, based on the complete BP measurements and the results of carotid ultrasonography.

The study protocol was reviewed and approved by the Ethics Committee of Beijing An Zhen Hospital, Capital Medical University. All participants provided the written informed consent.

### Data collection

We adopted a standard questionnaire designed by the World Health Organization MONICA project (Monitoring Trends and Determinants in Cardiovascular Disease) to investigate the personal information, medical history, smoking history, personal disease history, and family disease history of the participants. Demographic and anthropometric measures such as height, weight, and waist and hip circumference were obtained from all participants. Body mass index (BMI) was calculated as weight in kilograms divided by the square of height in meters.

BP was measured three times at 5-minute intervals with the use of a validated semi-automated electronic device (Omron HEM-770A; Omron Healthcare, Kyoto, Japan). Persons were seated quietly for at least 5 minutes in a chair, with feet on the floor, and arm supported at heart level. Caffeine, exercise, and smoking were avoided for at least 30 minutes prior to measurement. The three readings and the average systolic BP (SBP) and diastolic BP (DBP) were recorded by trained operators. Within-visit SBP and DBP variability were expressed as the maximum absolute difference (MAD) between any two readings of three measurements during one visit period. Hypertension was defined by mean of SBP ≥140 mmHg and/or mean of DBP ≥90 mmHg and/or use of antihypertensive drugs at the time of the survey.

Venous blood samples from each fasted participant were drawn following BP measurement. Serum glucose, total cholesterol (TC) and triglycerides (TG) were determined by standard enzymatic methods. High-density lipoprotein cholesterol (HDL-C) and low-density lipoprotein cholesterol (LDL-C) were measured by a homogeneous assay.

### Carotid ultrasonography

The carotid ultrasound examination was performed using an ALOKA Prosound α10 (ALOKA Medical, Japan) with a 7.5 MHz linear array transducer. Trained sonographers performed ultrasound scans on the participants in a supine position with the head slightly extended and turned to the opposite direction of the carotid artery being studied. The images were recorded at 12 different carotid sites (i.e., right and left, near and far walls, common, bifurcation and internal carotid artery) according to a standardized protocol. The assessment of intima media thickness (IMT) of each carotid segment (i.e., common, bifurcation and internal carotid artery) was considered the mean of maximum IMT values of the four sites (i.e., right and left, near and far walls) in regions free of plaque. The mean of the maximum IMT of the carotid bifurcations and the common carotid arteries was used to designate increased IMT in the present analysis, and an increased IMT was defined as IMT ≥1 mm [14]. A Plaque was defined as IMT ≥1.3 mm [15] or a focal structure that encroaches into the arterial lumen of at least 0.5 mm or 50% of the surrounding IMT value [16], both of which were based on the single thickest wall in each arterial segment.

The carotid ultrasound examination was conducted by seven sonographers. All ultrasound scans were sent to the ultrasound reading center and manually analyzed by a single reader who underwent initial training and certification, with analyses performed using a GE EchoPAC digital reading station. To assess the reproducibility of IMT measurements, we selected two sonographers from seven operative doctors using random sampling to reexamined five participants at 1-week interval after the initial visit. The intra-sonographer intraclass correlation (ICC) was 0.98 and the inter-sonographer ICC was 0.94. To assess the reproducibility of the IMT imaging analysis, 20 imaging sequences were randomly sampled to be analyzed twice by the same reader and also analyzed once by another same level reader. The within-reader ICC was 0.96 and the between-reader ICC was 0.88.

### Statistical analyses

Data analyses were performed using SPSS 13.0 statistical software (Version 13.0; SPSS, Chicago, IL, USA). Continuous variables are expressed as mean ± standard deviation (SD), and categorical variables are described in frequencies or percentages. Group comparisons were performed using Student's *t*-test or median test or chi-square test as appropriate. We calculated the MAD between any two readings as an indicator of within-visit BP variability, and within-visit MAD of BP was divided into three groups according to mean plus or minus standard deviation (i.e., MAD ≤ mean − SD, mean – SD < MAD ≤ mean + SD, MAD > mean + SD) for further analysis. To examine the relative influence of within-visit BP variability on increased IMT and carotid plaque, multiple logistic regression analyses were performed, in which the SBP and DBP were respectively entered into separate models to avoid their collinear relationship. The odds ratio (OR) and 95% confidence interval (CI) of within-visit MAD of BP for increased IMT and carotid plaque were calculated. In all analyses, a value of *P*<0.05 was accepted as indicating statistical significance.

## Results

### Characteristics of study population

A total of 1222 subjects was included in this study, 45.5% of whom were male. The mean age of the participants was 65.2±8.0 years. Overall, the mean SBP and DBP were 136.8±17.3 and 82.3±9.6 mmHg, and the prevalence rate of hypertension was 61.9%. Of the hypertensive patients, 44.4% were undergoing treatment with antihypertensive drugs. Within-visit MAD of BP was represented as the parameter of BP variability in this study. In total, the range of within-visit MAD in SBP was 0–52 mm Hg (median, 10.0 mm Hg; 25th to 75th percentiles, 6.0–15.0 mm Hg), and the range of within-visit MAD in DBP was 0–34 mm Hg (median, 5.0 mm Hg; 25th to 75th percentiles, 3.0–8.0 mm Hg). The characteristics of participants among the three groups of within-visit SBP variability (MAD ≤ mean −SD, mean – SD < MAD ≤ mean + SD, MAD > mean + SD) showed that age, BMI, TG, fasting glucose, mean SBP, DBP level, the prevalence of smoking, diabetes, hypertension and antihypertensive medication tended to increase significantly with an increase in within-visit SBP variability (Table 1). The characteristics of participants among the three groups of within-visit DBP variability showed similar results.

**Table 1:** Characteristics of study population among three groups of within-visit SBP variability (MAD≤mean-SD, mean-SD<MAD≤mean+SD, MAD>mean+SD).

Characteristics	Total	Within-visit MAD in SBP			*P* for trend
		≤4	5–18	>19	
Number of subjects, n	1222	192	857	173	
Age, y	65.2±8.0	64.2±8.4	65.1±8.0	66.4±7.8	<0.01
Smoking,%	15.2	20.3	15.3	9.2	<0.01
Body mass index, kg/m^2^	24.58±3.17	23.95±3.17	24.61±3.15	25.12±3.18	<0.001
Total cholesterol, mmol/L	5.36±1.01	5.27±1.00	5.36±1.00	5.46±1.05	0.08
Triglycerides, mmol/L	1.80±1.19	1.73±1.24	1.76±1.16	2.03±1.22	0.02
HDL-C, mmol/L	1.33±0.26	1.31±0.25	1.34±0.26	1.33±0.25	0.33
LDL-C, mmol/L	3.31±0.88	3.23±0.81	3.32±0.90	3.35±0.90	0.17
Fasting glucose, mmol/L	5.90±1.04	5.69±0.81	5.89±0.97	6.19±1.47	<0.001
Diabetes,%	9.6	6.3	9.1	15.6	<0.01
Mean of SBP, mmHg	136.8±17.3	128.6±17.0	137.0±16.7	145.0±16.0	<0.001
Mean of DBP, mmHg	82.3±9.6	79.7±9.8	82.3±9.3	85.6±9.8	<0.001
Hypertension,%	61.9	51.0	61.3	76.9	<0.001
Antihypertensive medication,%	44.4	38.5	43.8	54.3	<0.01
Heart rate, beats/min	71.6±11.1	72.3±11.5	71.1±10.7	73.1±12.7	0.56
Within-visit MAD in SBP					
Range, mm Hg	0–52	0–4	5–18	19–52	
Median (IQR), mm Hg	10.0 (6.0, 15.0)	3.0 (1.0, 4.0)	10.0 (7.0, 14.0)	22.0 (20.0, 26.0)	<0.001*
Mean (SD), mm Hg	11.2±7.1	2.3±1.5	10.6±3.7	24.0±5.7	<0.001
Within-visit MAD in DBP					
Range, mm Hg	0–34	0–17	0–29	0–34	
Median (IQR), mm Hg	5.0 (3.0, 8.0)	4.0 (2.0, 6.0)	6.0 (4.0, 8.0)	8.0 (5.0, 11.0)	<0.001*
Mean (SD), mm Hg	6.2±4.1	4.4±3.7	6.3±3.9	8.1±4.9	<0.001

Abbreviations: BP, blood pressure; SBP, systolic blood pressure; DBP, diastolic blood pressure; MAD, maximum absolute difference; HDL-C, high-density lipoprotein cholesterol; LDL-C, low-density lipoprotein cholesterol; SD, standard deviation; IQR, interquartile range.

*Median test.

### Prevalence of increased IMT and carotid plaque with incremental within-visit BP variability

**Figure 1:**
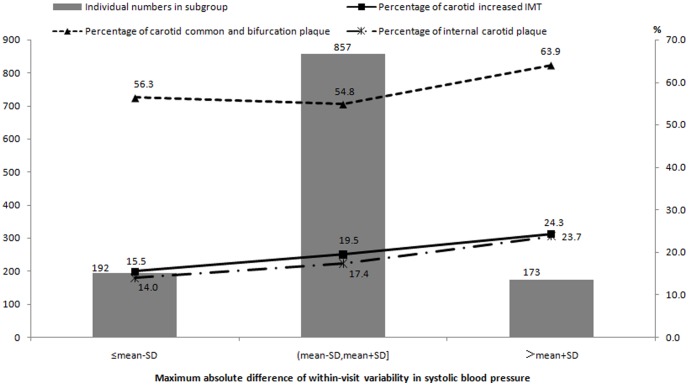
Prevalence of carotid increased IMT and plaque stratified by within-visit maximum absolute difference of systolic blood pressure overall.

**Figure 2:**
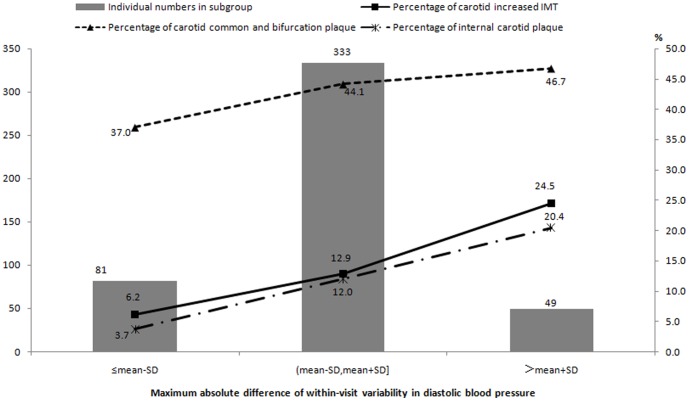
Prevalence of carotid increased IMT and plaque stratified by within-visit maximum absolute difference of diastolic blood pressure in normotensive subjects.

**Figure 3:**
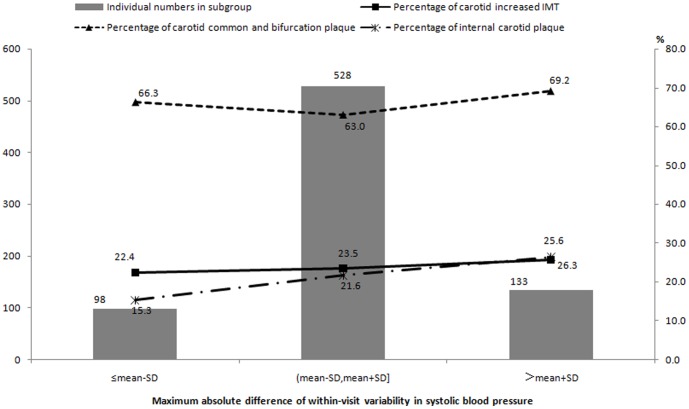
Prevalence of carotid increased IMT and plaque stratified by within-visit maximum absolute difference of systolic blood pressure in hypertensive patients.

In the overall population, the prevalence rates of increased IMT were 15.5%, 19.5%, and 24.3% in the three groups of within-visit SBP variability, and the prevalence rates of internal carotid plaque were 14.0%, 17.4%, and 23.7% in the three groups of within-visit SBP variability; both of these rates had an increased tendency with increasing within-visit MAD of SBP (*P*_trend_ = 0.04 and *P*_trend_ = 0.02, Figure 1). When we categorized this study population into normotensive subjects and hypertensive patients, we found a difference between the two subpopulations. Among normotensive subjects, the prevalence rates of increased IMT and internal carotid plaque increased significantly with incremental within-visit MAD of DBP (both *P*_trend_  = 0.003, Figure 2), however, among hypertensive patients, an increasing trend was found in the prevalence rate of internal carotid plaque with incremental within-visit MAD of SBP (*P*_trend_  = 0.05, Figure 3).

### Association of within-visit BP variability with the risk of having increased IMT and carotid plaque

After adjustment for age, sex, smoking, BMI, TC, TG, HDL-C, LDL-C, fasting glucose, heart rate, and mean BP, multiple analyses showed no significant effects of within-visit MAD in SBP or DBP on the risk of having increased IMT and plaque in the overall population (Table 2). Further subgroup analyses were conducted in the normotensive and hypertensive population, which showed some differences between the two subgroups. In the normotensive participants, the subjects with the highest within-visit DBP variability (i.e., MAD of DBP > mean + SD) had 4.92 times the odds of having increased IMT (OR = 4.92, 95%CI: 1.48–16.42, *P* = 0.01) and 6.07 times the odds of internal carotid plaque (OR = 6.07, 95%CI: 1.31–28.10, *P* = 0.02) in comparison with those participants with the lowest within-visit DBP variability (i.e., MAD of DBP ≤ mean −SD, Table 3). However, in the hypertensive patients, within-visit SBP variability was significantly associated with having internal carotid plaque: the subjects with MAD of SBP > mean + SD had 2.86 times the odds of having internal carotid plaque in comparison with those with MAD of SBP ≤ mean − SD (OR = 2.86, 95%CI: 1.16–7.04, *P* = 0.02; Table 4). When we subdivided hypertensive patients into subpopulations with treatment or without treatment, this result was only found to remain significantly in hypertensive patients who were on antihypertensive drugs therapy (OR = 3.54, 95%CI: 1.26–10.00, *P* = 0.02; Table 5).

**Table 2:** Odds ratios for risk of carotid increased IMT and having plaque by maximum absolute difference of within-visit variability in blood pressure overall.

MAD of within-visit variability in blood pressure	Increased IMT		Common and bifurcation carotid plaque		Internal carotid plaque	
	OR (95% CI)	*P* value	OR (95% CI)	*P* value	OR (95% CI)	*P* value
SBP, mmHg						
≤mean-SD	1.00		1.00		1.00	
(mean-SD, mean+SD]	1.33 (0.83–2.13)	0.23	0.76 (0.52–1.10)	0.14	1.00 (0.57–1.75)	1.00
>mean+SD	1.58 (0.88–2.84)	0.13	0.89 (0.54–1.46)	0.64	1.70 (0.85–3.40)	0.14
DBP, mmHg						
≤mean-SD	1.00		1.00		1.00	
(mean-SD, mean+SD]	1.35 (0.85–2.12)	0.21	1.21 (0.84–1.75)	0.30	1.42 (0.81–2.48)	0.22
>mean+SD	1.60 (0.89–2.87)	0.12	1.31 (0.80–2.16)	0.28	1.37 (0.65–2.88)	0.41

Abbreviations: MAD, maximum absolute difference; SBP, systolic blood pressure; DBP, diastolic blood pressure; SD, standard deviation; IMT, intima-media thickness; OR, odds ratio; CI, confidence interval.

Adjusted variables included age, sex, smoking, BMI, TC, TG, HDL-C, LDL-C, fasting glucose, heart rate, and mean systolic blood pressure level.

**Table 3:** Odds ratios for risk of carotid increased IMT and having plaque by maximum absolute difference of within-visit variability in blood pressure in normotensive subjects.

MAD of within-visit variability in blood pressure	Increased IMT		Common and bifurcation carotid plaque		Internal carotid plaque	
	OR (95% CI)	*P* value	OR (95% CI)	*P* value	OR (95% CI)	*P* value
SBP, mmHg						
≤mean-SD	1.00		1.00		1.00	
(mean-SD, mean+SD]	1.67 (0.71–3.93)	0.24	0.66 (0.39–1.13)	0.13	0.64 (0.27–1.49)	0.30
>mean+SD	2.31 (0.68–7.86)	0.18	0.60 (0.25–1.44)	0.26	0.62 (0.16–2.36)	0.48
DBP, mmHg						
≤mean-SD	1.00		1.00		1.00	
(mean-SD, mean+SD]	2.27 (0.83–6.23)	0.11	1.19 (0.68–2.07)	0.55	3.27 (0.87–12.28)	0.08
>mean+SD	4.92 (1.48–16.42)	0.01	1.13 (0.50–2.56)	0.77	6.07 (1.31–28.10)	0.02

Abbreviations: MAD, maximum absolute difference; SBP, systolic blood pressure; DBP, diastolic blood pressure; SD, standard deviation; IMT, intima-media thickness; OR, odds ratio; CI, confidence interval.

Adjusted variables included age, sex, smoking, BMI, TC, TG, HDL-C, LDL-C, fasting glucose, heart rate, and mean systolic blood pressure level.

**Table 4:** Odds ratios for risk of carotid increased IMT and having plaque by maximum absolute difference of within-visit variability in blood pressure in hypertensive patients.

MAD of within-visit variability in blood pressure	Increased IMT		Common and bifurcation carotid plaque		Internal carotid plaque	
	OR (95% CI)	*P* value	OR (95% CI)	*P* value	OR (95% CI)	*P* value
SBP, mmHg						
≤mean-SD	1.00		1.00		1.00	
(mean-SD, mean+SD]	1.25 (0.70–2.23)	0.44	0.92 (0.55–1.55)	0.76	1.38 (0.64–2.99)	0.41
>mean+SD	1.44 (0.72–2.85)	0.30	1.16 (0.61–2.20)	0.65	2.86 (1.16–7.04)	0.02
DBP, mmHg						
≤mean-SD	1.00		1.00		1.00	
(mean-SD, mean+SD]	1.15 (0.67–1.99)	0.61	1.28 (0.78–2.09)	0.33	1.04 (0.54–2.02)	0.90
>mean+SD	1.09 (0.55–2.17)	0.81	1.45 (0.76–2.74)	0.26	0.77 (0.31–1.90)	0.57

Abbreviations: MAD, maximum absolute difference; SBP, systolic blood pressure; DBP, diastolic blood pressure; SD, standard deviation; IMT, intima-media thickness; OR, odds ratio; CI, confidence interval.

Adjusted variables included age, sex, smoking, BMI, TC, TG, HDL-C, LDL-C, fasting glucose, heart rate, mean systolic blood pressure level and antihypertensive drugs.

**Table 5:** Odds ratios for risk of carotid increased IMT and having plaque by maximum absolute difference of within-visit variability in blood pressure in hypertensive patients with antihypertensive therapy.

MAD of within-visit variability in blood pressure	Increased IMT		Common and bifurcation carotid plaque		Internal carotid plaque	
	OR (95% CI)	*P* value	OR (95% CI)	*P* value	OR (95% CI)	*P* value
SBP, mmHg						
≤mean-SD	1.00		1.00		1.00	
(mean-SD, mean+SD]	1.38 (0.69–2.73)	0.36	1.04 (0.57–1.88)	0.90	1.32 (0.56–3.09)	0.53
>mean+SD	1.50 (0.66–3.44)	0.34	1.72 (0.81–3.68)	0.16	3.54 (1.26–10.00)	0.02
DBP, mmHg						
≤mean-SD	1.00		1.00		1.00	
(mean-SD, mean+SD]	1.30 (0.67–2.53)	0.44	1.31 (0.74–2.34)	0.36	1.08 (0.50–2.33)	0.84
>mean+SD	1.12 (0.48–2.62)	0.80	1.65 (0.76–3.58)	0.21	0.91 (0.31–2.69)	0.86

Abbreviations: MAD, maximum absolute difference; SBP, systolic blood pressure; DBP, diastolic blood pressure; SD, standard deviation; IMT, intima-media thickness; OR, odds ratio; CI, confidence interval.

Adjusted variables included age, sex, smoking, BMI, TC, TG, HDL-C, LDL-C, fasting glucose, heart rate, and mean systolic blood pressure level.

We have also analyzed the association between within-visit BP variability and carotid IMT as continuous variable in multiple logistic regression models. After adjusting for conventional cardiovascular risk factors including mean SBP, the results showed that in hypertensive patients with antihypertensive therapy, the β of within-visit MAD in SBP for carotid IMT was 0.001, but had not reached statistical significance (P = 0.092), and in normotensive subjects or in hypertensive patients without antihypertensive therapy, there was no significant relationship between within-visit SBP or DBP variability and carotid IMT as continuous variable. We considered that the reason might be that the precision of IMT was so high that the effects could not be discriminated. The results in this section were not described in the tables.

## Discussion

In the present study, clinic sitting BP was measured three times within one visit and carotid arteries were scanned by B-mode ultrasonography for the detection of early atherosclerosis. MAD of three interval measurements was calculated and used as an indicator of within-visit BP variability. The results suggested that within-visit DBP variability was associated with increased IMT and internal carotid plaque in normotensive subjects, and within-visit SBP variability was associated with internal carotid plaque in hypertensive patients, especially in hypertensives undergoing antihypertensive treatment.

BP is a highly variable physiologic trait, its fluctuations being the result of a complex interaction between environmental stimuli and the response of cardiovascular control mechanisms [8], [9], [17], [18], [19], [20]. BP variability can be classified into long-term and short-term variability, the latter being characterized by spontaneous variation within a 24-hour period. Compared with long-term variability, short-term BP variable indicators were easier to measure and collect. Previous studies mostly focused on exploring the association between short-term BP variability derived from 24-hour ambulatory BP monitoring and target organ damage [15], [21], and reported that BP variability derived from several clinic visits or 24-hour ambulatory monitoring were better predictors of cardiovascular events when compared with within-visit BP variability at one follow-up [6]. However, by contrast with visit-to-visit BP variability, within-visit BP variability expressed as the MAD of three readings taken at one visit was little affected by the change of life style and the intervention of other risk factors, which was more likely a vascular compliance response resulting from sympathetic nervous activation or an alteration of baroreflex function. In addition, within-visit BP variability was more easily available than 24-hour ambulatory or visit-to-visit variability of BP.

The results from the Anglo-Scandinavian Cardiac Outcomes Trial Blood Pressure Lowering Arm (ASCOT-BPLA) showed that BP variability was a stronger predictor of stroke and coronary events than mean blood pressure [5], [6]. In ASCOT-BPLA, within-visit SBP variability, which was expressed as the SD and range of the three readings, was reported as a predictor of cardiovascular events [5]. Nagai et al. found that exaggerated visit-to-visit BP fluctuations were significant indicators for carotid artery atherosclerosis independently of average BP, which added evidence of the association between visit-to-visit variability in BP and subclinical carotid atherosclerosis [22]. In the present study, we provided evidence on the subclinical level that within-visit BP variability was also associated with increased IMT and internal carotid plaque, independent of the impacts of mean BP and heart rate on the carotid arteries. The epidemiological studies suggested that BP variability should not be neglected since mean BP, though important, could not account for all blood-pressure-related risk of cardiovascular diseases and the full benefit of antihypertensive drugs [5], [6]. Both increased mean BP and variability in BP can increase intravascular pressure or shear force, additionally causing endothelial function damage and promoting the development of atherosclerosis [10], [11]. Experimental studies in animals showed that increased short-term BP variability caused end-organ damage at a normal mean BP, consistent with the evidence in humans [5], [6]. Our study demonstrated that within-visit BP variability had significant relevance to subclinical damage of carotid arteries, although the direction of this relationship could not be determined. Because a large MAD might be due to increased arterial stiffness (larger BP changes in response to changes in stroke volume) or may also be linked with alterations in arterial baroreflex owing to reduced carotid compliance, we suggest that within-visit BP variability should not be neglected while measuring the mean BP in clinical patients.

This study found that within-visit BP variability during a single visit was related to increased IMT and internal carotid plaque among both normotensives and treated hypertensives. Our study suggested that the potential risks of residual BP variability among treated hypertensive patients, which were proved to account partly for the risk of subclinical carotid atherosclerosis, should not be ignored. In untreated hypertensive patients, the subjects with the highest within-visit SBP variability (i.e., MAD of SBP > mean + SD) had 2.09 times the odds of having internal carotid plaque in comparison with those with the lowest within-visit SBP variability (i.e., MAD of SBP ≤ mean – SD), but it did not reach statistical significance (OR = 2.09, 95%CI: 0.21–21.34, *P* = 0.53). We considered that the lack of effect in untreated subjects might be due to smaller group size, because only 39 subjects with the highest within-visit SBP variability (i.e., MAD of SBP > mean + SD) in untreated hypertensive patients. By contrast, the link between within-visit BP variability and carotid atherosclerosis in normotensives had only been previously reported. In earlier studies, it was reported that even within the normal range, antihypertensive drugs also reduced blood-pressure-related risk for adverse cardiovascular outcomes [23], [24]. In addition, the results from recently published reports showed that patients with consistently normal SBP (stable normotension) experienced very few vascular events, whereas those with normal mean SBP but high variability were at increased risk [6]. Coupled with our findings, the current evidence supports the notion that reductions in variability, rather than reductions in mean BP, might account for the benefits of antihypertensive drugs in subjects with normal BP.

This study has some limitations. First, it was a cross-sectional survey, so the link between BP variability within one visit and carotid atherosclerosis was unable to deduce a causal sequential conclusion. Second, our study was unable to eliminate the impact of the “white-coat effect” (WCE) on within-visit BP variability. On analyzing the large MAD of SBP for variable patterns, the results showed that 50% of the first measurements were the highest, whatever in the overall population or in hypertensive patients. The similar results were represented for MAD of DBP. We considered that although within-visit BP variability could not exclude the WCE and that both likely resulted from sympathetic nervous activation, the WCE could not be used as a surrogate for within-visit BP variability since it did not appear to be a manifestation of all within-visit BP (SBP or DBP) variability. Despite this, we still were able to conclude that within-visit BP variability during one follow-up period, whether caused by WCE or not, is associated with carotid atherosclerosis.

## Conclusions

Our study proposed that within-visit BP variability based on three interval measurements during a single visit has an important role in the evaluation of early atherosclerosis. Clinicians should be aware of the prognostic implications of BP variability within one clinic visit, and methods to improve its clinical application are worthy of further research.
